# The Blood Pressure "Uncertainty Range" – a pragmatic approach to overcome current diagnostic uncertainties (II)

**DOI:** 10.1186/1468-6708-6-5

**Published:** 2005-04-06

**Authors:** Cornel Pater

**Affiliations:** 1 Chippenham, UK

## Abstract

A tremendous amount of scientific evidence regarding the physiology and physiopathology of high blood pressure combined with a sophisticated therapeutic arsenal is at the disposal of the medical community to counteract the overall public health burden of hypertension. Ample evidence has also been gathered from a multitude of large-scale randomized trials indicating the beneficial effects of current treatment strategies in terms of reduced hypertension-related morbidity and mortality.

In spite of these impressive advances and, deeply disappointingly from a public health perspective, the real picture of *hypertension management *is overshadowed by widespread diagnostic inaccuracies (underdiagnosis, overdiagnosis) as well as by treatment failures generated by undertreatment, overtreatment, and misuse of medications.

The scientific, medical and patient communities as well as decision-makers worldwide are striving for greatest possible health gains from available resources.

A seemingly well-crystallised reasoning is that comprehensive strategic approaches must not only target hypertension as a pathological entity, but rather, take into account the wider environment in which hypertension is a major risk factor for cardiovascular disease carrying a great deal of our inheritance, and its interplay in the constellation of other, well-known, modifiable risk factors, i.e., attention is to be switched from one's "blood pressure level" to one's absolute cardiovascular risk and its determinants. Likewise, a risk/benefit assessment in each individual case is required in order to achieve best possible results.

Nevertheless, it is of paramount importance to insure generalizability of ABPM use in clinical practice with the aim of improving the accuracy of a first diagnosis for both individual treatment and clinical research purposes. Widespread adoption of the method requires quick adjustment of current guidelines, development of appropriate technology infrastructure and training of staff (i.e., education, decision support, and information systems for practitioners and patients). Progress can be achieved in a few years, or in the next 25 years.

## Introduction

During the past decades, *hypertension*, denoting abnormal elevation of blood pressure, has commonly been assigned a distinct *disease *quality. The majority of the medical community as well as renowned medical textbooks have considered it a pathological entity requiring diagnostic and appropriate treatment in most individuals having it.

Inherent in the 100-year old approach of discriminating between normal and abnormal blood pressure is, however, an arbitrary threshold established currently at 140/90 mmHg for *mild *hypertension and two higher cut-off values to define *moderate *and *severe *hypertension. This classification, still maintained as such by the ESH [1] and WHO [2] guidelines, has been in use for many decades both for management of hypertension in individual subjects and for defining patient population samples targeted for testing of new antihypertensive drugs.

It is common knowledge, however, that up to 95% of hypertensive individuals have high blood pressure of unknown etiology denoted *essential hypertension*. The majority of these have mild hypertension while some 10% of them have either moderate or severe degrees of hypertension.

Classically, the diagnosis of essential hypertension has been regarded as pretty straightforward, as long as a *secondary hypertension *could be ruled out with confidence.

While this latter entity can, on justifiable grounds, be considered a disease, essential hypertension is a quantitative expression of the fluctuations of a biologic variable – the blood pressure – that should be considered a major risk factor for cardiovascular disease at best, rather than a disease by itself.

Nevertheless, with increasing awareness that the trade-off between normality and abnormality on a blood pressure curve is confounded by large diurnal and random variation of the blood pressure variable, by individual factors such as age, sex, race as well as by a great number of potential errors occurring during blood pressure measurement *per se*, the medical and scientific community are desperately in search for approaches to increase the accuracy of diagnosis and management of hypertension and to shift the weight of decision making from the current "BP-value"-focused attitude to the appraisal of any patient's absolute risk, rather than his/her individual risks (i.e., high blood pressure, hypercholesterolemia, smoking, overweight, etc.) [3-5].

The driver behind the need for radical change is at least two fold:

1. The widespread awareness that the hypertension-related risk – in terms of cardiovascular complications, stroke and renal disease – rises in relation to increases of both systolic and diastolic blood pressure [6-13].

2. The hypertension diagnosis currently implies not only exclusion of secondary causes of hypertension but as well, careful consideration is to be given to differentiating entities like: *white coat hypertension *[14-17], *white coat effect *[18,19], *masked hypertension *[20-22] and *prehypertension*, from the genuine, sustained hypertension [23].

The clustering of these entities around the current diagnostic cut-off point (140/90 mmHg) generates a BP *uncertainty range *(130/85-160/95 mmHg) reflecting a universally widespread inaccuracy of a first diagnosis based on office BP measurements and a consequent inappropriate long-term management of the subjects assessed.

## Blood pressure measurement as a diagnostic test – a clinical epidemiology perspective

Commonly, variation in clinical medicine may be due to fluctuations of biologic variables or the presence or absence of disease as well as the nature of that disease and its severity; it may also be due to differences in measurement technique, errors in measurement, observer bias, and to a great extent to random variation.

The blood pressure variable happens to be a typical example that displays all the aforementioned variation parameters. An attempt to understand this variation through gaining insight in relative simple epidemiology and statistical concepts and then acting on the basis of the new acquired knowledge, might be the key to account for the blood pressure overall variability, to accurately interpret the prognostic significance of any blood pressure values and confidently manage pharmacological therapy in all hypertensive patients.

**Figure 1:**
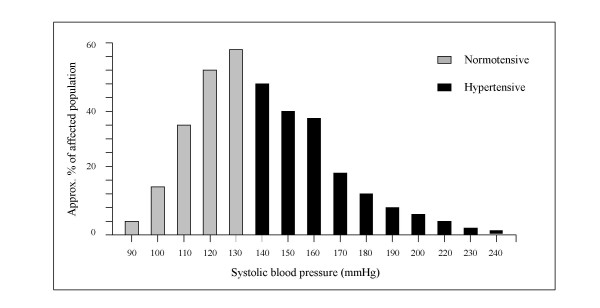
Normal distribution of SBP values in the general population.

**Figure 2:**
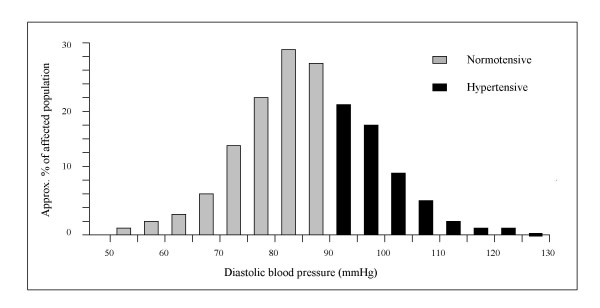
Normal distribution of DBP values in the general population.

In the general population blood pressure values follow a smooth bell-shaped distribution as displayed in Fig. 1 for the SBP and in Fig. 2 for the DBP. The two histograms attempt to account for the known general prevalence of hypertension worldwide [24], with higher figures for the SBP as compared with DBP due to their divergent pattern at higher ages (and both sexes) [25]. The black bars highlight the frequency distribution of the blood pressure values considered abnormal (according to the arbitrary threshold of 140/90 mmHg).

The distribution of SBP is slightly skewed to the right due to the high proportion of people with BP values in the range 140-160 mmHg [26].

**Figure 3:**
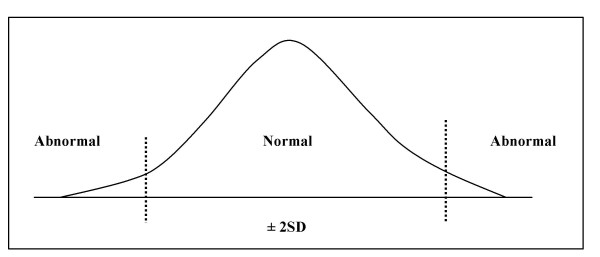
Illustration of the *normality *concept in a normal distribution.

Certainly, both systolic and diastolic blood pressure belong to one and the same, simplified, theoretical normal distribution (Fig. 3) assumed to describe the underlying population from which relevant data might be derived [27,28]. Fig. 3 suggests that all normal blood pressure values measured in the population are comprised by the interval given by the mean ± two standard deviations (M ± 2SD).

Assuming that the commonly used office blood pressure measurement was an ideal test (a "gold standard", which is not the case), it could separate all healthy people from those who have the disease (i.e., people with BP values that require treatment from those who do not). In such a hypothetical case the blood pressure measurement would be 100% sensitive and 100% specific, with no false-positive or false-negative results.

In reality, things are much different; there are two normal distributions of blood pressure "test" results. One is for individuals free of disease and one for individuals who have the disease. Framing the reality in this way is still misleading because in practice many patients may have raised blood pressure levels (but are "disease free", insofar as they may not need pharmacologic treatment – e.g., white coat hypertension) [29,30] and, many patients may display apparently normal blood pressure values but, definitely need pharmacologic treatment (because of high absolute risk for CVD, the presence of a compelling indication or of masked hypertension) [29,30].

**Figure 4:**
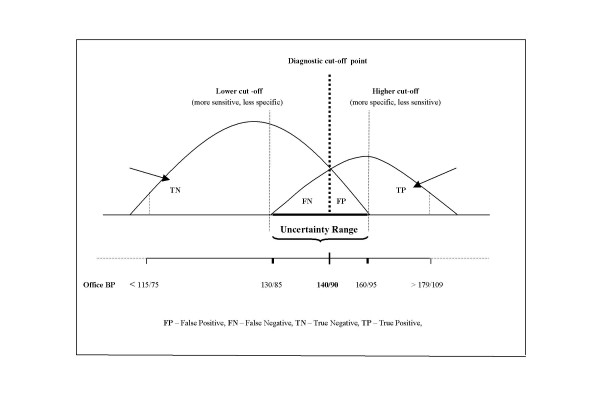
Result alternatives by office BP measurement and the trade-off displayed by the sensitivity and specificity of the method as related to the cut-off point selected (140/90 mmHg).

In fact, there is an overlap of the two distributions and that overlap is rather large; it predisposes to both over- and underdiagnosis, i.e., false-positive and false-negative results. The immediate consequence under the current circumstances of conventional office BP measurement is that setting any test value (i.e., BP threshold) as cut-off point to distinguish between "normal" and "abnormal", will misclassify a great proportion of the patients falling into the overlap area (Fig. 4) – a real *uncertainty range *exposing to misdiagnosis (i.e., under- and overdiagnosis) [30].

As Fig. 4 illustrates, for our diagnostic "test" (the office BP measurement) which does not behave as a "gold standard", there are four distinct alternatives [31]:

1. True negatives (TN) – subjects without the disease who test negative (i.e., normal BP and no indication for blood pressure lowering treatment).

2. True positives (TP) – subjects who have the disease and test positive (i.e., sustained hypertension requiring pharmacologic treatment).

3. False-negatives (FN) – subjects who have the disease but test negative (i.e., normal BP, however, in need to get pharmacologic treatment (e.g., masked hypertension).

4. False-positives (FP) – subjects who do not have the disease but test positive (i.e., subjects with high blood pressure values but in no need for pharmacologic treatment (e.g., white coat hypertension).

Fig. 4 also illustrates the dynamic complexity of what goes on around the cut-off point selected. As mentioned in the first part of this paper, many national hypertension societies still maintain a cut-off point for blood pressure normality/abnormality by 160/95 mmHg [32]. For those societies which decided to select 140/90 mmHg as cut-off point, the switch from 160/95 to 140/90 mmHg has meant a substantial change of several important diagnostic parameters (including the prevalence of the condition) [33].

Namely, the sensitivity of the test has increased, however, with a simultaneous decrease in its specificity. The number of TPs has increased as compared to the number of FNs. Further, the move of the cut-off point to the left on the BP curve has caused an increase in FPs as compared to the FNs.

With other words, a high cut-of point on the BP curve implies less false-positive results (and less overdiagnosis) with the reverse occurring if the cut-off point is low.

**Figure 5:**
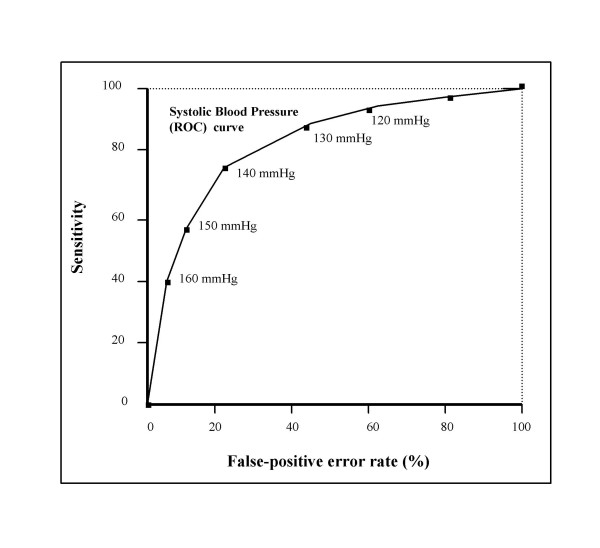
Receiver operating characteristic curve exploring the proper cut-off point for blood pressure measurement.

Selection of "best cut-off point" can be enhanced by constructing a receiver operating characteristic (ROC) curve (Fig. 5) [34]. Such a curve displays *sensitivity *on the X-axis and the *false positive error rate *(1-specificity) on the Y-axis. These two parameters can be computed for different threshold values on the basis of points plotted on the graph, as results of blood pressure measurements in individuals with known health status (hypertension/no hypertension).

A cut-off point set at 0 would mean a "test" (i.e., BP measurement) with 100% sensitivity and ability to detect all patients with genuine hypertension (i.e., sustained hypertension). However, all normal individuals (i.e., without hypertension) would screen positive, as reflected by a 100% false-positive error rate (overdiagnosis). The corresponding point would be placed in the right upper corner of the graph.

In the other extreme, a hypothetical cut-off point of 350 mmHg, for example, would imply that virtually all hypertensive patients were missed (underdiagnosis), reflected by 0% sensitivity. This point would be placed in the lower left corner of the graph.

Using a similar reasoning, the sensitivity and the false-positive error rate can be computed for increasing threshold values. Connecting the points plotted on the graph would generate a ROC curve.

As the left upper corner represents a sensitivity of 100% and 0% false-positive rate, the real best cut-off point is the one lying closest to it.

Fig. 5 suggests that the 140/90 mmHg is, as a matter of fact, the best cut-off point, a fact that should satisfy all those who are sceptical as to the value of this particular threshold value. Obviously, its use does not preclude a refined physician-patient dialog aiming at accounting for the patients view regarding a particular antihypertensive treatment, as Campbell and Murchie put it: "Appropriate management of blood pressure should be guided by an informed dialogue between patients and doctors and not by blind pursuit of blood pressure targets"[35].

In fact, despite the drawbacks mentioned above, of an arbitrarily selected office blood pressure cut-off point for practical purposes, the office BP measurement as a method as such, need not be entirely discarded.

On the contrary, there are three different instances in which it may be used with certainty to decide whether to treat or not to treat patients. The first two instances are discernable from Fig. 4 and pertain to individuals without any *compelling indication *(i.e.., diabetes mellitus or renal dysfunction):

1). The great majority of individuals with office BP > 160/95 mmHg are genuine hypertensives. They may have *sustained *essential hypertension (or a secondary form of hypertension) [36]. Approximately 50% of myocardial infarctions and one third of strokes are known to be associated with BP > 160/95 mmHg. The presence of one or several other risk factors further increase the certainty that these patients need pharmacological treatment and careful, long-term follow-up [5,37].

2). Likewise, the great majority of individuals with office BP < 130/80 mmHg are likely to be free of hypertension and/or the need for pharmacologic blood pressure lowering treatment [38]. Regular health checks, for example at two years intervals, are likely to capture propensity toward increased risk, particularly among individuals who, according to the current classification belong to the *high-normal *prehypertension category (130/85-139/89 mmHg).

In this context, it is worth re-emphasizing that blood pressure values below the 140/90 mmHg cut-off point do not confer total protection against a cardiovascular events. Data from Framingham Study have shown that more than one half (57%) of all heart attacks and almost one half of all strokes in some population studies occur in persons with normal office blood pressure [39-41].

3). The third instance pertains to patients with known diabetes mellitus and renal dysfunction. For these patients, the office blood pressure measurement functions merely as a quasi-"gold standard" test.

**Figure 6:**
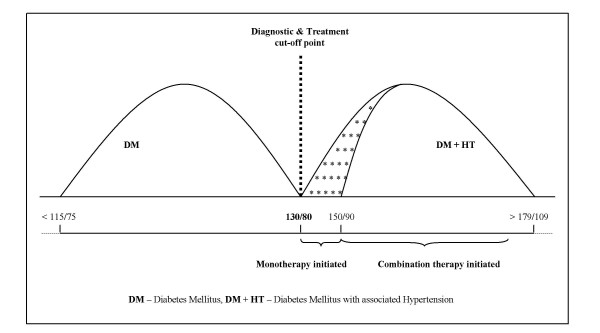
Hypothetical, non-overlapping distributions of diabetics and diabetics with associated hypertension patients.

Fig. 6 illustrates the concept of two population distributions: one of diabetics without hypertension and another of diabetics with associated hypertension. Given the widespread consensus that patients with diabetes and associated hypertension should get pharmacological treatment whenever BP > 130/80 mmHg [42,43], the two distributions do not appear to have any degree of overlap, with the cut-off point of 130/80 mmHg discriminating well between those supposed to be treated pharmacologically, from those who are not.

A justifiable question that may arise is why a somewhat lower cut-off point, i.e., 130/80 mmHg, can be considered as more reliable than the 140/90 mmHg, given that office BP measurement is likely to carry the same sort of problems in both cases.

The answer is pretty simple. An assumed overlap of the two distributions (Fig. 6), leading to FP assessments (overdiagnosis) and FNs (underdiagnosis), would cause neither diagnosis nor management concerns. Namely, patients with diabetes and associated hypertension need aggressive antihypertensive treatment meant to lower BP as much as possible [44-47]. Blood pressure measurement errors around the cut-off point value of 130/80 mmHg are, therefore, likely to have neither clinical nor prognostic relevance, as long as the diabetes mellitus diagnosis is certain.

Furthermore, Fig. 6 depicts a second cut-off point relevant for the population of diabetics with associated hypertension: the 150/90 mmHg, which, according to current guidelines is an indication for use of combination therapy (two drugs from the start).

**Figure 7:**
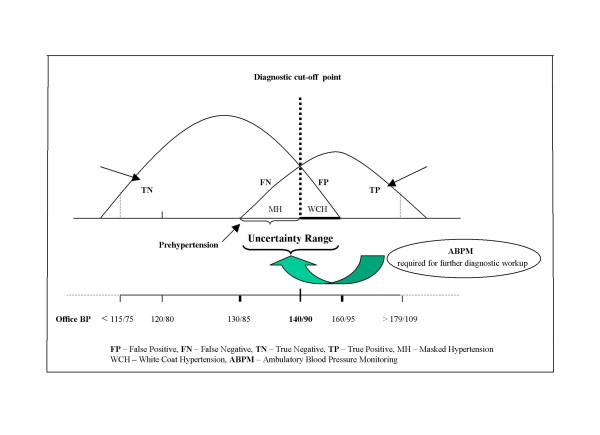
The *uncertainty range *and its mix of hypertension categories. Accurate diagnosis is only possible by ABPM.

At closer scrutiny of the *uncertainty range *(≥ 130/85 to <160/95 mmHg) (see Fig. 7) from its lower bound upward, as assessed by office blood pressure measurements, it appears to be a mix of populations consisting of:

• Subjects with prehypertension (*high-normal *BP ≥ 130/85-139/89 mmHg)

• Patients with masked hypertension

• Subjects with white coat hypertension

• Patients with sustained hypertension

**Figure 8:**
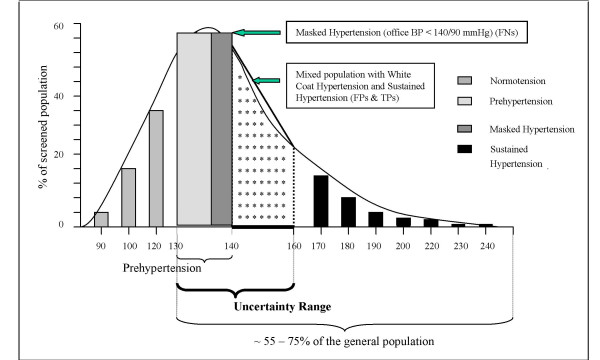
Frequency distribution of hypertensive categories as parts of a common, normal distribution.

Fig. 8 attempts to depict the frequency distribution of the aforementioned patient categories in a single, common population distribution. It suggests that a great part of the total population lies either in the in the *uncertainty range *or under the area consisting of genuine hypertensive patients (i.e., with sustained hypertension).

A recent analysis of the worldwide global burden of hypertension [48] indicated that 26.4% of the adult population in 2000 had hypertension and that 29.2% were projected to have the condition by 2025. The hypertension prevalence is, however, considerably much higher in economically developed countries as compared with economically developing countries (37.3% versus 29.9%) [48].

A multinational sample surveys carried out in six European countries, Canada and US, in the 1990s indicated the age-adjusted prevalence of hypertension was 28% in the North American countries and 44% in the European countries [24].

About 59 million American adults (29%) fall into prehypertension category (SBP 120-139 mmHg or DBP 80-89 mmHg) [49].

Extrapolating roughly from the above figures, the prevalence of hypertension in economically developed countries (37.3%) and the estimation of prehypertension in US (29%) would add up to a 66.3% of the entire population having a form of hypertension or prehypertension. This average figure might be somewhat lower in America and Canada but may reach 75% in the European countries.

Furthermore, epidemiological data [50] indicate that approximately 25% of the community burden of BP-related CVD is occurring among the population of hypertensives with systolic BP ≥ 160 mmHg (representing only approximately 5% of the total number of hypertensives). About 33% of all BP-related CVD events are likely to occur in the persons within normotensive BP range (<140 mmHg) while more than 66% of the same burden can be attributed to the patient population with systolic BP ≥ 140 mmHg, i.e., belonging to the *uncertainty range*. Given that the lower bound of the *uncertainty range *extends to 130/85 mmHg (to include the *high-normal *prehypertension), it might encompass as much as 80% of the total community burden of BP-related CVD risk.

## Prehypertension (PH)

The new JNC-7-hypertension category [49] was subject to heavy criticism, primarily by European scientists who argued that the new entity might have negative psychological impact and generate a wave of more or less unnecessary investigations in fairly healthy individuals.

This is, certainly, not a strong argument even if it may contain a grail of truth. Obviously, the sudden and unexpected awareness that one, despite knowing him/herself to be in good health, belong to a group of people "at risk for heart disease", might trigger a more or less uncomfortable feeling.

However, people with BP values in the range of 130-139/85-89 mmHg, known to have *high normal *blood pressure and labelled as being *prehypertensives*, are also known to run a considerable higher risk for cardiovascular disease than people with *optimal *(<120/80 mmHg) and with *normal *BP (120-129/80-84 mmHg) [49,51,52].

In a survey of 9845 Framingham Heart Study participants over a 4-year period, 43% of the total of 1907 individuals (19% of the original sample) who had *high-normal *blood pressure at the initial screening have developed hypertension [53]. In contrast, only 6% of the subjects with *optimal *blood pressure and 20% of those with *normal *blood pressure have developed hypertension [53]. Hypertension incidence in all three categories increased with age, reaching 37% among subjects with *high-normal *BP who were 35-64 years old and 50% among subjects aged 65 or older, likewise, with *high-normal *blood pressure at screening. Worthwhile emphasizing, about 15% of the subjects with *high-normal *blood pressure have progressed to stage II or greater degree of hypertension over 4 years of follow-up.

Overall, compared with *optimal *blood pressure, *high-normal *blood pressure was associated with a 5- (age 35-64 years) to 12-fold (age 65 years or over) elevated odds of hypertension on follow-up [53], justifying inclusion of these subjects in the uncertainty area and thereby, the consequent need for accurate diagnosis from the outset as well as appropriate long-term management of these individuals.

## Masked Hypertension (MH)

As depicted by Fig. 7, individuals with MH fall into the *false negative *area of the uncertainty range, suggesting that the condition *per se *is perceived as normotension as assessed by office blood pressure measurement. However, MH is genuine hypertension, as assessed by daytime ABP of >135/85 mmHg [54-58].

The reported prevalence rates of MH are 9% [59], 14% [60], 23% [61] and 31% [62]. Individuals with masked hypertension were shown to be similar with true hypertensive patients in terms of left ventricular characteristics, carotid artery wall thickness, and prevalence of discrete atherosclerotic plaques [63] but to be different on several demographic and lifestyle variables (greater proportion of males, older, greater degree of alcohol consumption, past smoking) [60,63].

Missed diagnosis of MH in these patients leaves them untreated and at risk for long-term consequences of hypertension.

Selenta et al. [60] have demonstrated that the BP values falling in the borderline range (10 mmHg above and below 140/90 mmHg) are particularly inaccurate. Most participants presumed healthy in the 10 mmHg range below 140/90 mmHg have hypertensive ABP, i.e., MH, commonly missed by the office BP measurement. The authors of the same article concluded that only those office readings averaging 20 points above or below the 140/90 mmHg cut-off, represent safe diagnostic information.

The high prevalence rates of MH and the high level of misdiagnosis rate by office BP measurement of the condition calls for generalization of ABPM use in clinical practice, for diagnosis and management purposes of this large patient population.

## White coat hypertension (WCH)

Subjects with white coat hypertension have a normal average daytime blood pressure outside a medical setting [64,65] but present with high BP in the medical environment [66,67]. By definition [68,69], white coat hypertension is diagnosed as such, if the conventional BP is persistently ≥ 140/90 mmHg and the average daytime ambulatory BP is below 135/85 mmHg.

The prevalence rate of the WCH is reported to be in the area of 15 to 35% of patients in whom hypertension is diagnosed [70,71] and in nearly 30% of pregnant women [72].

The use of a distinct cut-off point is important for diagnostic and management purposes. It also distinguishes WCH from the *white coat effect*, the latter being a quantitative measure of the blood pressure rise in the presence of a physician. This transient blood pressure rise has been quantified by Mancia et al. [73] who demonstrated mean value increases of 27 mmHg for both systolic and diastolic pressure when measurement was done in the presence of a physician. The white coat effect is commonly defined as an office BP exceeding mean daytime ambulatory BP by at least 20 mmHg systolic and/or 10 mmHg diastolic [74]. Such a large white coat effect has been found in as many as 73% of treated hypertensive subjects and it may occur more frequently in women than in men [75,76].

As illustrated by Fig. 7, both WCH as a distinct entity and the white coat effect as a transitory BP rise, fall into the *false positive *area of the *uncertainty range*, i.e., beyond the 140/90 mmHg cut-off point as assessed by office BP measurement, and count thereby as overdiagnosis.

Indeed, the consequences of failing to identify white coat hypertension are considerable. People may be penalized for insurance and pension policies, and for employment [77].

Long-term treatment may be prescribed unnecessarily with all the risks derived from potential treatment-emergent adverse reactions [78-80].

Given the seriousness as well as the high risk for such consequences in the clinical practice, several hypertension guidelines recommend use of ABPM for diagnosis of WCH [81-83].

In 2001, the CMS in USA has selected "patients with suspected WCH" as having indication for ABPM, with the use of method itself being reimbursed [84].

Speculations on what clinical characteristics might suggest the presence of WCH and thereby the need for ABPM, in an attempt to preclude indiscriminate use of the method, is certainly not justified anymore [85]. On the contrary, current evidence argues in favour of ABPM use in all patients with office blood pressure falling in the *uncertainty range *(≥ 130/85 to <160/95 mmHg).

Once WCH has been diagnosed, ABPM should be repeated at annual or biannual intervals [86] with the aim to capture increased cardiovascular risk that would justify initiation of drug treatment [87,88].

## A holistic approach toward hypertension management

**Figure 9:**
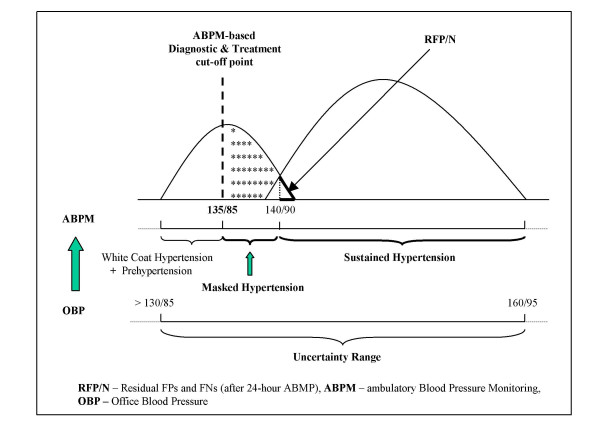
ABPM assessment of subjects with BP values falling in the *uncertainty range*.

Fig. 7 suggests that ABPM should be used for further diagnostic work up of subjects whose office-measured BP values fall in the *uncertainty range*. Indeed, combining office blood pressure (OBP) measurement values with ABPM recording results (daytime normality cut-off point of 135/85 mmHg) allows for discrimination among the following diagnostic entities (see Fig. 9):

• Sustained Hypertension (SH) – (ABPM > 140/90 and OBP ≥ 140/90)

• Masked Hypertension (MH) – (ABPM > 135/85 and OBP < 140/90)

• White Coat Hypertension (WCH) – (ABPM < 135/85 and OBP ≥ 140/90)

• Prehypertension (*high-normal *BP) (PH) – (ABPM < 135/85 and OBP 130/85-139/89)

The specific thresholds used for diagnostic discriminatory purposes can also be used for management purposes. It is widely agreed that poor control of hypertension is defined by BP values > 140/90 mmHg [89] while good control, in terms of ABPM, counts ≤ 135/85 mmHg [90-92]. Likewise, there is widespread agreement that patients with clinic pressures of = 160/95 mmHg need drug treatment [93].

The well-known, multifactorial inaccuracies imbedded in the office blood pressure measurement and, in contrast to that, the widespread agreement as to the superiority and multi-purpose use of ABPM [94-102], as well as reports on its cost-effectiveness [103] [104], make the method to emerge as an additional alternative to the conventional approach of hypertension management. While ABPM can, on no account, be a replacement for the conventional office blood pressure measurement, it is increasingly obvious that its use is a *sine qua non *condition for accurate diagnosis and management of subjects belonging to a large segment of the general population displaying different forms of high blood pressure-related entities (falling in the *uncertainty range*).

More recent studies provide evidence that the ABP testing is generally well-accepted and tolerated by patients. A survey of 177 patients, who underwent ABPM in primary care office setting in US over a 2.5 years period [103], showed that 75% of them considered the 24-hour ABPM test as worthwhile, with respect to the time and money incurred by the investigation. Ninety per cent of the patients thought that the results of the test were to provide useful information for the physician's decision making on appropriate therapy. Similar results were derived from a qualitative study of ABPM in UK [71]. Both studies have emphasized, however, the importance of the explanation given by the physician to the patient as to the benefit of undergoing ABPM testing, in order to minimize the perception of discomfort related to the 24-hour recording.

**Figure 10:**
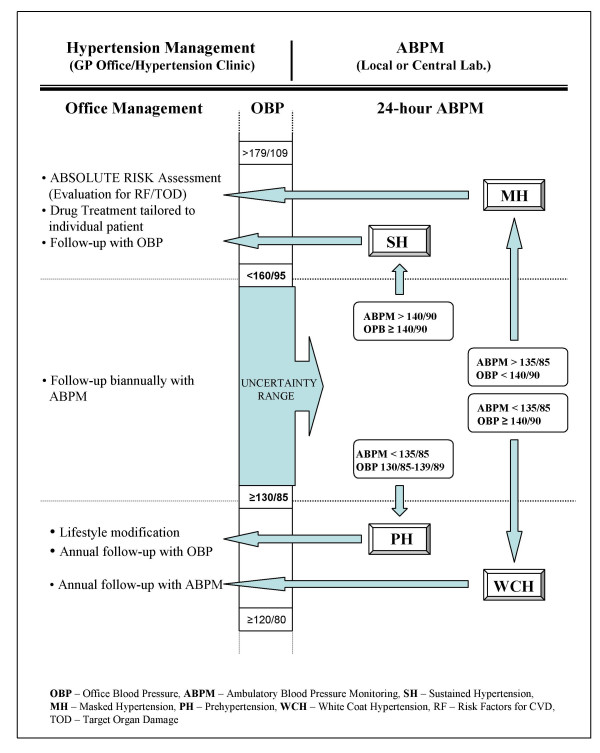
An algorithmic approach to management of subjects with OBP in the *uncertainty range *(≥ 130/85 to <160/95 mmHg).

Fig. 10 is an algorithmic approach to the management of all subjects who display office blood pressure falling in the *uncertainty range *(≥ 130/85 to < 160/95 mmHg). The top part of the figure suggests that two different units are involved in the management/investigation of such patients, a usual GP or specialist office and a laboratory specialised in ABPM recordings and results interpretation.

An initial assessment is commonly performed in the GP's office where blood pressure is recorded, according to current guidelines, by the physician or a trained nurse. Patients whose average BP, derived from two measurements per visit, in two or several visits, lies in the uncertainty range should be referred for 24-hour ABP monitoring.

The result of the daytime ABPM assessed together with the OBP generates a diagnostic conclusion matching one of the following alternatives: SH/MH/WCH or PH (*high-normal *level).

Amazingly, the current conventional cut-off point of 140/90 mmHg looses entirely its value for decision making, at least in the initial stage assessment.

Indeed, as mentioned above, much information may be lost when sensitivity and specificity are defined in relation to a single cut-off point value of a continuous variable (such as the BP). Instead, using a range, i.e., the interval between two cut-off points (≥ 130/85 to <160/95 mmHg) for further decision making, avoids the well-known diagnostic uncertainties.

The 140/90 cut-off point retains, however, its (arbitrary) value in the next stage of ABPM investigation. The value of the OBP, whether below or above the 140/90 mmHg threshold is corroborated with the ABPM result for selection of one out of four different diagnostic alternatives: sustained hypertension, masked hypertension, white coat effect or *high-normal *prehypertension.

Once diagnosed, the patient returns to his/her physician who remains in charge with the further management decision whatever the diagnosis might be (including lifestyle changes, drug treatment, follow-up, etc.).

Obviously, diagnosis and management of patients who have any form of symptomatic atherosclerotic vascular disease including previous myocardial infarction, by pass graft surgery, angina, stroke or transient ischaemic attack, peripheral vascular disease or atherosclerotic renovascular disease, need treatment of even mild hypertension (≥ 140/90 mmHg) for *secondary prevention*. Likewise, patients with target organ damage such as LVH, heart failure, proteinuria or renal impairment need treatment of even very mild hypertension.

Patients with type I and II diabetes mellitus and associated mild hypertension (≥ 130/80 mmHg) generally have diabetic nephropathy and should be treated.

All the aforementioned *compelling indications *require drug treatment for any level of raised blood pressure.

This translates in the need to apply the algorithm in Fig. 10 and perform formal absolute risk assessment only in patients with uncomplicated hypertension (i.e., with BP values falling in the *uncertainty range *≥ 130/85 to <160/95 mmHg).

## Conclusion

Accurate diagnosis and management of high blood pressure is of paramount importance for the prevention of long-term, cardiovascular, cerebrovascular and renal complications. Aggressive attempts to identify and treat "high blood pressure values" must be balanced carefully with the risks of overdiagnosis and overtreatment in these patients.

ABPM has a proven value not only as a research tool but also as a valuable investigative method for a large segment of the hypertensive population belonging to the *uncertainty range*.

Practical management of these patients, once accurate diagnosis has been established, should be based on absolute cardiovascular disease risk and on a risk-communication dialog between the physician and patient as well as on their mutual agreement regarding the specific treatment to be initiated and the appropriate long-term follow-up.

## Competing interests

The author(s) declare that they have no competing interests.
